# Proteomics Analysis of Celiac Disease-Active Peptides in Food Products with Partially Hydrolyzed Gluten

**DOI:** 10.1021/acs.jafc.5c13719

**Published:** 2026-01-12

**Authors:** Eleonora Tissen, Lana Dakwar, Sabrina Geisslitz, Katharina Anne Scherf

**Affiliations:** † TUM School of Life Sciences, Professorship of Food Biopolymer Systems, 9184 Technical University of Munich, Lise-Meitner-Str. 34, Freising 85354, Germany; ‡ Leibniz Institute for Food Systems Biology at the Technical University of Munich, Lise-Meitner-Str. 34, Freising 85354, Germany

**Keywords:** barley malt extract, barley malt vinegar, immunogenic epitopes, nanoLC-MS/MS, soy sauce, stable isotope dilution assay

## Abstract

Foods with partially hydrolyzed gluten may still trigger immune responses in celiac disease (CeD) patients due to residual CeD-active peptides. We applied nanoliquid chromatography-tandem mass spectrometry (nanoLC-MS/MS) to identify 54 CeD-active peptides in 15 barley malt extracts (BME), seven barley malt vinegars (BMV), and 15 soy sauces. Eight common CeD-active peptides were quantitated with targeted nanoLC-MS/MS and stable isotope dilution assay. Total peptide concentrations ranged from 481 mg/kg to 1305 mg/kg in BMEs, whereas three samples showed peptide concentrations near LOQ. Six BMVs had peptide concentrations of up to 0.04 mg/kg, and in soy sauce, they ranged from 0.2 mg/kg to 0.5 mg/kg. Peptide concentrations correlated with gluten content measured by R5c ELISA in BMEs and BMV, while soy sauce showed a gluten content below the LOQ by ELISA. These findings show the presence of CeD-active peptides in foods containing partially hydrolyzed gluten, highlighting the need for comprehensive risk assessment.

## Graphical abstract

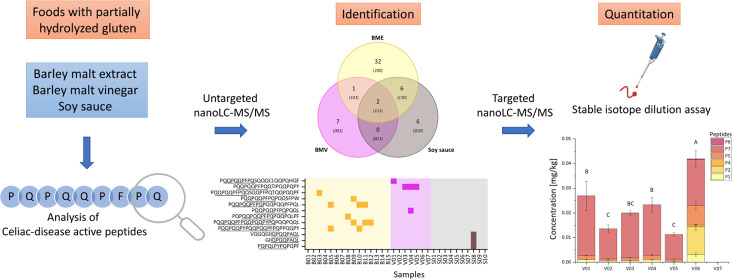

## 1 Introduction

Celiac disease (CeD) is a chronic immune-mediated disorder triggered by the ingestion of gluten, which is a heterogeneous protein mixture found in wheat (gliadins and glutenins), rye (secalins), and barley (hordeins).^1^ In genetically predisposed individuals, gluten-derived peptides are presented by human leukocyte antigen (HLA)-DQ2 or HLA-DQ8 receptors on antigen-presenting cells, leading to the activation of CD4^+^ T cells and subsequent inflammation and mucosal damage in the small intestine.^2^ These CeD-active peptides are resistant to gastrointestinal digestion and vary in length, but distinct core sequences of nine amino acids, the CeD-active epitopes, serve as a binding motif for HLA-DQ2/8.^3,4^

Currently, the only effective treatment is a strict, lifelong gluten-free diet for CeD patients. According to Regulation (EU) No 828/2014,^5^ products labeled “gluten-free” must not exceed a threshold of 20 mg/kg of gluten. A particular challenge arises from partially hydrolyzed gluten, as found in germinated, fermented or enzymatically treated foods such as barley malt products and soy sauce. For example, barley malt extracts (BME) are frequently used as flavoring or sweetening agents in foods that are not traditionally based on gluten-containing cereals. Even small amounts may introduce partially hydrolyzed gluten, creating uncertainties for CeD patients and complications regarding risk assessment.^6^ During food processing, intact gluten proteins are fragmented into heterogeneous peptides, of which only a subset carries CeD-active epitopes relevant for immune activation.^7^ However, limitations in current analytical methods hinder the reliable detection and quantitation of these remaining peptides. Gluten analysis relies on antibody-based assays, such as competitive enzyme-linked immunosorbent assays (cELISA), that recognize specific epitopes like QQPFP, QQQFP, LQPFP, and QLPFP.^8^ As a result, they may fail to detect CeD-active epitopes without ELISA-recognition epitope that contribute to the total gluten content and have immunostimulatory properties.^9,10^ Panda et al. (2017) demonstrated that multiplex cELISA applied for fermented and partially hydrolyzed foods like soy sauce produced nonspecific inhibitory responses, preventing antibodies from binding to potentially immunoreactive gluten peptides.^11^ Furthermore, the lack of suitable reference materials reflecting diverse proteolytic conditions during food processing makes the accurate analysis across different food products even more difficult.^12^

Liquid chromatography coupled with tandem mass spectrometry (LC–MS/MS) is a powerful complementary technique, enabling the specific detection of the remaining gluten sequences in foods containing partially hydrolyzed gluten.^13–15^ Most applications have focused on relative quantitation, particularly in beer, where peptide abundances are compared between samples based on relative signal intensities.^16^ Beyond that, Li et al. (2018) detected gluten in malt vinegar but not in soy sauce using LC–MS/MS.^17^ This approach enabled the detection of trace amounts of remaining potentially harmful epitopes. Nevertheless, no validated routine method currently exists for the absolute quantitation of CeD-active peptides in various fermented or partially hydrolyzed products.

Therefore, the objective of this study was to identify potentially remaining CeD-active peptides in the partially hydrolyzed, germinated and/or fermented food products BME, barley malt vinegar (BMV), and soy sauce with high resolution untargeted nanoLC-MS/MS. Moreover, we aimed to quantitate the absolute peptide concentrations of selected CeD-active peptides in these samples by targeted nanoLC-MS/MS with stable isotope dilution assay (SIDA). The ability to accurately quantitate trace amounts of potentially harmful gluten peptides provides critical data for future studies on immunoreactivity and improved risk management strategies for foods containing partially hydrolyzed gluten.

## 2 Materials and Methods

### 2.1 Reagents

All chemicals were of analytical or higher grade and purchased from VWR Merck (Darmstadt, Germany), Fisher Scientific (Waltham, MA, USA), Carl Roth (Karlsruhe, Germany), or Sigma-Aldrich (Steinheim, Germany).

### 2.2 Sample Collection

Eight dried BMEs (B01 to B08), four liquid BMEs (B09 to B12), and 15 soy sauces (S01 to S15) were obtained from international vendors in 2022. Industrial manufacturers provided gluten-free grain sorghum extract (Briess Malt & Ingredients, Chilton, WI, USA) and three liquid BMEs (B13 to B15). The seven BMVs (V01–V07) were purchased from vendors in the UK in 2023. Further sample information is provided in Table 1 if noticeable differences in, for example, sample consistency, color, or ingredients were visible.

**Table 1:** Sample Set of Different Fermented and Partially Hydrolyzed Food Samples (Barley Malt Extract, Barley Malt Vinegar, and soy Sauce), Analyzed for the Identification of Celiac Disease (CeD)-Active Peptides by Untargeted Nano-Liquid Chromatography-Tandem Mass Spectrometry^a^ ^,^ ^b^

ID	further sample information	R5c gluten content [mg/kg]	crude protein content [ %]	no. of identified gluten peptides	no. of identified peptides with R5 epitope	no. of identified peptides with CeD-active epitope
barley malt extract						
B01	powder, light color	3704.5 ± 371.8	5.7 ± 0.04	107	23	17
B02	powder, amber color	3871.8 ± 282.9	5.8 ± 0.04	128	26	18
B03	powder, dark color	3772.0 ± 260.1	5.6 ± 0.01	135	11	12
B04	powder, extra light color	3304.6 ± 82.4	5.3 ± 0.009	104	17	16
B05	powder, light color	2795.8 ± 129.4	5.0 ± 0.1	118	24	18
B06	powder, medium color	5416.6 ± 641.1	5.9 ± 0.01	99	24	21
B07	powder, dark color	4727.7 ± 388.3	5.4 ± 0.02	162	13	10
B08	liquid, syrupy, light color	4941.3 ± 126.1	5.0 ± 0.1	145	22	19
B09	liquid, syrupy, amber color	3936.4 ± 408.5	4.8 ± 0.05	152	25	15
B10	liquid, syrupy, dark color	3413.3 ± 227.5	4.5 ± 0.05	108	22	20
B11	liquid, syrupy, medium color	3488.0 ± 340.9	5.7 ± 0.06	80	14	11
B12	liquid, syrupy, medium color	4978.5 ± 386.4	4.8 ± 0.05	142	18	16
B13	liquid, dark color	11.7 ± 0.8	4.1 ± 0.06	41	n.d	2
B14	liquid, dark color	<LOQ	1.3 ± 0.06	9	2	n.d
B15	liquid, dark color, roasty malt extract	<LOQ	1.9 ± 0.04	16	1	1
barley malt vinegar						
V01		18.8 ± 2.7	0.40 ± 0.03	94	5	2
V02		16.9 ± 4.0	0.38 ± 0.02	86	2	n.d
V03		14.5 ± 1.5	0.38 ± 0.01	86	3	1
V04		17.3 ± 2.1	0.38 ± 0.02	96	8	5
V05		15.2 ± 3.0	0.36 ± 0.00	111	5	3
V06		25.5 ± 2.3	0.28 ± 0.01	57	3	4
V07	distilled	13.2 ± 1.8	0.037 ± 0.001	13	n.d	n.d
soy sauce						
S01	gluten-free	<LOQ	8.1 ± 0.03	2	n.d	1
S02		<LOQ	6.7 ± 0.04	6	n.d	2
S03		<LOQ	7.7 ± 0.01	3	n.d	n.d
S04		<LOQ	6.9 ± 0.00	9	1	1
S05		<LOQ	7.7 ± 0.05	4	n.d	1
S06		<LOQ	7.3 ± 0.2	5	1	n.d
S07		<LOQ	7.0 ± 0.2	5	n.d	1
S08		<LOQ	8.5 ± 0.04	52	3	6
S09		<LOQ	4.6 ± 0.1	6	n.d	n.d
S10		<LOQ	3.7 ± 0.07	7	n.d	n.d
S11	sweet	<LOQ	2.5 ± 0.08	6	n.d	1
S12	sweet, viscous	<LOQ	0.4 ± 0.01	40	13	7
S13	sweet, viscous	<LOQ	0.3 ± 0.005	17	4	1
S14		<LOQ	3.6 ± 0.1	4	n.d	n.d
S15		<LOQ	3.1 ± 0.08	18	11	5

a LOQ: limit of quantitation for the R5c ELISA is 10 mg/kg; No.: number; n.d.: not detected.

b Further sample information is given as provided by the manufacturers if differences in sample consistency, color, or ingredients were visible. The gluten content was analyzed by competitive R5 (R5c) enzyme-linked immunosorbent assay (ELISA), and crude protein content was analyzed with the Dumas combustion method and a calculation factor of 5.71. Values for gluten and crude protein content (*n* = 3) are given as means ± standard deviation. Total numbers are given for identified gluten peptides and peptides with at least one R5 epitope or CeD-active epitope.

### 2.3 Gluten and Crude Protein Content

The R5 Ridascreen Gliadin competitive ELISA kit (R5c ELISA) was purchased from R-Biopharm (Darmstadt, Germany), and samples were extracted with 60% ethanol (v/v) and analyzed in triplicate exactly according to the manufacturer’s instructions. R5c antibodies recognize specific epitopes from prolamin sequences (QQPFP, QQQFP, LQPFP, and QLPFP),^8^ and the resulting color intensity is inversely proportional to the analyte concentration defined by a calibration curve based on digested prolamins from wheat, barley and rye.^18,19^ Gluten content is derived after multiplication of the prolamin content with the factor 2, which relies on the assumption of a 1:1 prolamin-to-glutelin ratio in gluten.^20^ The nitrogen content of the samples was determined in triplicate by the Dumas combustion method ICC-Standard 167 (2000) using a Dumatherm Nitrogen analyzer (Gerhardt Instruments, Königswinter, Germany).^21^ Crude protein content was calculated using a factor of 5.71.

### 2.4 Sample Preparation for Untargeted Nanoliquid Chromatography-Tandem Mass Spectrometry

BME (50 mg) was stirred with tris­(hydroxymethyl)­aminomethane (Tris) solution (0.5 mL, 0.5 mol/L, pH 8.5 with 50% 1-propanol (v/v) and 2 mol/L urea) for 30 min at 60 °C. After the extraction step, the suspension was centrifuged for 25 min at 3550 rcf and 22 °C. The supernatant was evaporated to dryness and the residue was dissolved in Tris–HCl (0.5 mL, 0.5 mol/L, pH 8.5) and 1-propanol (0.5 mL). Reduction was performed by adding tris­(2-carboxyethyl)­phosphine (TCEP) (50 μL, 0.08 mol/L TCEP in 0.5 mol/L Tris–HCl with 16.7 mol/L urea, pH 8.5) and incubation for 30 min at 60 °C. Cysteine residues were alkylated with chloroacetamide (CAA) (0.1 mL, 0.4 mol/L CAA in 0.5 mol/L Tris–HCl with 16.7 mol/L urea, pH 8.5) for 45 min at 37 °C in the dark, followed by evaporation. Chymotryptic hydrolysis (0.5 mL, enzyme-to-substrate ratio 1:25, 0.1 mol/L Tris–HCl, 3.3 mol/L urea, pH 7.8) was performed for 18 h at 37 °C in the dark. The reaction was stopped with 5 μL trifluoroacetic acid. Sample cleanup was done with solid phase extraction (SPE) using Discovery DSC-18 SPE 96-well plates with a bed weight of 100 mg/well (Supelco, Sigma-Aldrich). The wells were activated with 2 mL of methanol and equilibrated with 2 mL of 80% (v/v) acetonitrile and 0.1% (v/v) formic acid in water. Afterward, a conditioning step was performed with 3 mL of 2% (v/v) acetonitrile and 0.1% (v/v) formic acid in water. The digested samples were loaded onto the wells and allowed to drip through without vacuum. Then, they were washed with 5 mL of 2% (v/v) acetonitrile and 0.1% (v/v) formic acid in water. The peptides were eluted without vacuum with 2 × 0.5 mL of 40% (v/v) acetonitrile and 0.1% (v/v) formic acid in water. The eluates were collected in 2 mL microtubes, and the solvent was evaporated to dryness. The residue was dissolved in 1 mL of 0.1% formic acid (FA) and diluted to achieve an approximate protein concentration based on the crude protein content of 200 ng/μL. Finally, the solution was filtered through a 0.45 μm polyvinylidene fluoride (PVDF) membrane (MilliporeSigma by Merck, Darmstadt, Germany).

Sample preparation of BMVs (0.5 mL) and soy sauces (0.1 mL) started with the addition of 0.5 mL Tris–HCl (0.5 mol/L, pH 8.5) and 1-propanol (0.5 mL), followed by all steps as described above for BME, beginning with the reduction step without prior evaporation.

### 2.5 Untargeted Nanoliquid Chromatography-Tandem Mass Spectrometry

A Vanquish Neo nano system (ThermoFisher Scientific), coupled to an Orbitrap Exploris 480 mass spectrometer (ThermoFisher Scientific), was used. Peptides were first loaded onto an Acclaim PepMap Neo C18 (5 μm, 300 μm × 5 mm) nanotrap column (ThermoFisher Scientific) and then separated with an Aurora Ultimate XT C18 (25 × 75 μm) UHPLC column (IonOpticks, Collingwood, Australia) under the following conditions: solvent A consisted of 0.1% formic acid in water; solvent B consisted of 0.1% formic acid in 80% acetonitrile with 20% water (v/v) with a flow rate at 300 nL/min at a column temperature of 40 °C. The following gradient was used: 0 min 3% B, 60 min 40% B, followed by an automatic column wash with 99% B for 5 min and automatic column equilibration. The eluate from the analytical column was ionized using an EASY-Spray ion source (ThermoFisher Scientific) in positive electrospray ionization (ESI^+^) mode using a source voltage of 1.7 kV at a capillary temperature of 275 °C. The MS/MS system was operated using data-dependent acquisition (DDA), selecting the 20 most intense precursor ions from each MS1 full scan for fragmentation with an isolation width of *m*/*z* 2.0 at 30% normalized collision energy and default charge state of 2+. MS1 spectra were obtained at a resolution of 120,000 (at *m*/*z* 200) within a mass range of *m*/*z* 375–1200, with an automatic gain control (AGC) target of 2 × 10^6^ and an automatic maximum injection time mode. MS2 spectra were additionally acquired at a resolution of 15,000 for ions with charges ranging from 2+ to 5+, with an AGC target of 5 × 10^4^, an automatic maximum injection time mode, and a fixed first mass of *m*/*z* 120. The dynamic exclusion was set to 30 s. Data acquisition was performed with Xcalibur (ThermoFisher Scientific, version 4.2.47).

### 2.6 Peptide Identification

Peptides were identified with the software MaxQuant (version 2.6.2.0).^22^ The MS raw data were directly searched as input against three FASTA files of the UniProtKB.^23^ They contained all entries with the accession as protein family, protein name or gene name “hordein” (taxonomy: [4513], 80 results, downloaded August 2025), “glutenin” (taxonomy: [4565], 1713 results, downloaded August 2025) and “gliadin” (taxonomy: [4565], 1774 results, downloaded August 2025) to focus on all possible gluten peptides from barley and wheat. The parameters were set as follows: digestion mode: specific; enzyme: chymotrypsin+ (cleavage after phenylalanine, tryptophane, tyrosine, leucine and methionine); maximum missed cleavage sites: 2; variable modifications: oxidation (M), acetyl (protein N-term); fixed modification: carbamidomethyl (C); minimum peptide length: 7; max. peptide mass: 4600 Da; 1% peptide and protein false discovery rate. Match between runs was activated, and the matching time window was set to 0.4 min. Other parameters were set as default and included, e.g., a search for potential contaminants.

Data analysis was performed using the MaxQuant-generated peptides.txt output table with Microsoft Office Excel 2016 (Microsoft Corporation, Seattle, WA, USA). Peptides identified as potential contaminants or with an Andromeda search score of 0 or intensity of 0 were excluded. The remaining peptides were classified as identified gluten peptides. To identify peptides with at least one binding site of the R5 monoclonal antibody, all identified peptide sequences were filtered for the main epitope sequences recognized by the R5 (QQPFP, QQQFP, LQPFP, and QLPFP) according to the literature.^8^ Furthermore, peptides were filtered to select sequences containing at least one complete CeD-active epitope according to Sollid et al. (2020) and Chlubnová et al. (2023).^3,4^ In the following, the CeD-epitopes are named according to Sollid et al. (2020),^4^ where hor-, glia-, and glut-are the short terms denoting the gluten protein of origin: hordein (in barley), gliadin, and glutenin (in wheat).

### 2.7 Standard Peptides

The CeD-active peptides (P1–P9; Table 2) for absolute quantitation with SIDA were selected based on the identified CeD-active peptides in the samples. The selection contained eight peptides with at least one CeD-active epitope identified across different fermented and partially hydrolyzed samples, thus representing a wide range of remaining CeD-active peptides. Unlabeled (standard peptides P1– P9) and stable isotope-labeled peptides (internal standards IS1–IS9) (Table 2) were synthesized by GenScript (Rijswijk, Netherlands). IS1–IS8 contained one fully [^13^C]- and [^15^N]-labeled amino acid (P or F). Peptides were solubilized according to the manufacturer’s guidelines for preparing stock solutions (1 mg/mL in water) and stored at −80 °C prior to use.

**Table 2:** Nano-Liquid Chromatography-Tandem Mass Spectrometry Parameters (Precursor Ions, Product Ions, and Corresponding Collision Energies (CE), Orbitrap Resolution and Maximum Injection Times (Max. IT)) for the Quantitation of Celiac Disease (CeD)-Active Peptides (P) by Targeted MS2, Including Abbreviations for P and Stable Isotope Labelled Internal Standards (IS), Amino Acid Sequences, the Respective CeD-Active Epitope According to Sollid et al. (2020) after Reversion of Deamidation^a^ ^,^ ^b^

P/IS	amino acid sequence	DQ2.5 restricted epitopes	protein accession (mass [Da])	conversion factor	precursor *m*/*z*	product ions *m*/*z*	CE [eV]	resolution	max. IT [ms]
P1	PQQPIPQQPQPY	hor-3a	I6TEV5 (33,456)	23.6	710.8646++	564.3 (b5+), 917.5 (b8+), 1142.6 (b10+), 857.4 (y7+), 504.2 (y4+)	15	60,000	118
IS1	PQQPIPQQPQ*PY				713.8715++	564.3 (b5+), 917.5 (b8+), 1142.6 (b10+), 863.4 (y7+), 510.3 (y4+)			
P2	PQQPQPFPQQPIPQQPQPY	hor-3a	I6TEV5 (33,456)	14.9	748.3796+++	823.4 (b7+), 1176.6 (b10+), 1067.6 (y9+), 857.4 (y7+), 504.2 (y4+)	15	45,000	86
IS2	PQQPQPFPQQPIPQQPQ*PY				750.3842+++	823.4 (b7+), 1176.6 (b10+), 1073.6 (y9+), 863.4 (y7+), 510.3 (y4+)			
P3	PQQPQPFPQQPIPQQPQPYPQQPQPF	hor-3a	I6TEV5 (33,456)	10.9	1022.5138+++	579.3 (b5+), 823.4 (b7+), 1176.6 (b10+), 1386.7 (b12+), 488.3 (y4+)	25	60,000	118
IS3	PQQPQPFPQQPIPQQPQPYPQQPQP*F				1025.8562+++	579.3 (b5+), 823.4 (b7+), 1176.6 (b10+), 1386.7 (b12+), 498.3 (y4+)			
P5	PQQPQQPFPQPQQPFPW	glia-γ4c, glia-y1a (DQ8), ^sec–1a^, hor −1^a^, glia-ω1^a^, glia-ω2	Q41210 (34,308)	16.5	1038.0103++	951.5 (b8+), 1176.6 (b10+), 1368.7 (y11+), 1124.6 (y9+), 899.4 (y7+)	16	90,000	182
IS5	PQQPQQPFPQPQQPF*PW				1041.0172++	951.5 (b8+), 1176.6 (b10+), 1374.7 (y11+), 1130.6 (y9+), 905.5 (y7+)			
P6	PQQPIPQQPQPYPQQPQPF	hor-3a	I6TEV5 (33,456)	14.9	1122.0658++	564.3 (b5+), 917.5 (b8+), 1402.7 (b12+), 1326.6 (y11+), 841.4 (y7+)	15	45,000	86
IS6	PQQPIPQQPQPYPQQPQP*F				1127.0794++	564.3 (b5+), 917.5 (b8+), 1402.7 (b12+), 1336.7 (y11+), 851.4 (y7+)			
P7	PQQPQQPFPQPQQPF	glia-γ4c, glia-y1a (DQ8), ^sec–1a^, hor −1^a^, glia-ω1^a^	Q41210 (34,308)	19.2	896.4443++	951.5 (b8+), 1176.6 (b10+), 1085.5 (y9+), 841.4 (y7+), 616.3 y5+)	22	30,000	56
IS7	PQQPQQPFPQPQQP*F				901.4579++	951.5 (b8+), 1176.6 (b10+), 1095.6 (y9+), 851.4 (y7+), 626.3 y5+)			
P8	SQQPIPQQPQPY	hor-3a	P17992 (8045, fragment)	5.7	705.9++	441.2 (b4+), 554.3 (b5+), 857.4 (y7+), 632.3 (y5+), 504.2 (y4+)	15	60,000	118
IS8	SQQPIPQQPQ*PY				708.9++	441.2 (b4+), 554.3 (b5+), 863.4 (y7+), 638.3 (y5+), 510.3 (y4+)			
P9	QPQQPFPQPQQPFPL	sec-1^a^, hor −1^a^, glia-ω1^a^	Q41210 (34,308)	19.3	889.0++	726.36 (b6+), 951.5 (b8+), 1304.6 (b11+), 826.4 (y7+), 473.3 (y4+)	16	60,000	118
IS9	QPQQPFPQPQQPF*PL				892.0++	726.36 (b6+), 951.5 (b8+), 1304.6 (b11+), 832.5 (y7+), 479.3 (y4+)			

a The corresponding protein accession numbers and their average masses without signal peptide are given for calculation of conversion factors used for calculation of the estimated gluten content.

b *P: proline ([13C]­5, 15N); *F: phenylalanine (1s9, 15N) aSequences are identical after reversion of deamidation; the following short terms only denote the proteins of origin. hor: hordein; glia: gliadin; sec: secalin.

### 2.8 Sample Preparation for Targeted Nanoliquid Chromatography-Tandem Mass Spectrometry

The sample preparation for the absolute quantitation of CeD-active peptides in the samples was based on the sample preparation for untargeted analysis as described above, with slight changes. BME (10 mg) was stirred with Tris solution (0.1 mL, 0.5 mol/L, pH 8.5 with 50% 1-propanol and 2 mol/L urea) for 30 min at 60 °C. After the extraction step, the sample was evaporated to dryness, and the dried sample was dissolved in Tris–HCl (0.1 mL, 0.5 mol/L, pH 8.5) and 1-propanol (0.1 mL). For the sample preparation of BMV (0.5 mL) and soy sauce (0.1 mL), 0.5 mL each of Tris–HCl (0.5 mol/L, pH 8.5) and 1-propanol was added. Additionally, a mixture of IS1–IS9 (10–40 μL) was added to the samples prior to digestion (BME, BMV and soy sauce) to achieve an analyte:IS ratio of 1 by adjusting the concentration of each IS. All the following steps were as described above, except that volumes were adjusted to one-fifth for BME, because 10 mg were used as starting material instead of 50 mg.

### 2.9 Response Lines for Calibration

Solution 1 with only light peptides P1–P9 and solution 2 with only IS1–IS9 were prepared from the stock solutions (1 mg/mL) in 0.1% (v/v) formic acid in water for calibration. The concentrations of P1–P9 were similar to the corresponding IS1–IS9. However, every individual peptide varied between 4 ng/mL and 200 ng/mL, depending on the expected concentration of every single peptide in the samples based on preliminary experiments. Afterward, solutions 1 and 2 were mixed with variable volumes to achieve different molar ratios *n*(P)/*n*(IS) between 5.1 and 0.2 (5 + 1, 4 + 1, 3 + 1, 2 + 1, 1 + 1, 1 + 2, 1 + 3, 1 + 4, 1 + 5) for calibration. The response lines were reanalyzed weekly, and a representative response line is shown in Figure S3.

### 2.10 Targeted Nanoliquid Chromatography-Tandem Mass Spectrometry

The same nanoLC-MS/MS system was used for targeted analysis with the same LC and ESI conditions described above for untargeted analysis, with a different gradient: 0 min 3% B, 40 min 40% B, followed by an automatic column wash with 99% B for 5 min and automatic column equilibration. The MS/MS system was operated in scheduled targeted MS2 mode with an isolation window of m/z 0.7, standard AGC target of 1 × 10^5^, default charge state of 2+, and a scan range of m/z 120–1500. An inclusion list with the accurate m/z of the peptides and their experimentally optimized collision energies, Orbitrap resolutions, maximum injection time, and resolution was employed (Table 2). Data acquisition was performed with Xcalibur (ThermoFisher Scientific, version 4.7.69.37).

### 2.11 Absolute Peptide Quantitation with Stable Isotope Dilution Assay

Data analysis was performed with the software Skyline (MacCoss Lab Software, University of Washington, Seattle, WA, USA, version 24.1.0.414)^24^ as described by Geisslitz et al. (2020)^25^ with slight changes. The transitions for each peptide were manually analyzed to assess the signal quality, overlapping transitions at the same chromatographic retention time, and m/z values. The precursor (2+, 3+, or 4+) with the highest peak area was taken. The five most abundant product ion transitions were selected based on the intensity. For that, at least one product ion m/z higher than the precursor m/z, and only transitions for product ions larger than y_2_-and b_2_-fragments were considered. The ratios between the five transitions were confirmed to be the same for the response lines and the samples (±5% absolute variation). The manual inspection of the peaks and intensities had to indicate correct peptide identification. The averaged peak ratio from the five transitions was used to calculate analyte concentrations. Response lines (*R* ^2^ > 0.99) were plotted by linear regression of the peak area ratios A­(P1–P9)/A­(IS1–IS9) against the respective molar ratios n­(P1– P9)/n­(IS1–IS9) from three replicates with one injection each. The absolute peptide concentrations for BMV and soy sauce, which were not weighed, were adjusted to the densities obtained from the FAO/Infoods Density Database (1.12 g/mL)^26^ and the mean value for commercial vinegars (1.02 g/mL).^27^

### 2.12 Precision

Samples B01, V02, and S02 were used to evaluate precision, because they contained many of the analyzed CeD-active peptides and were therefore considered as representative samples. Repeatability was evaluated by analysis of six replicates from the same starting material and prepared in parallel (*n* = 6) and intermediate precision with six replicates each prepared on 3 days (same analyst, same instrument), respectively (*n* = 18), according to Geisslitz et al. (2020).^25^ Precision was only calculated for the analytes that were detected in those samples. Additionally, precision was calculated as the Horwitz ratio (HorRat), a normalized performance parameter indicating the acceptability of analysis methods with respect to reproducibility.^28^ It is calculated by the predicted relative standard deviation (RSD) with the concentration (c) as mass fraction (1 μg/g = 1 × 10^–6^) and the experimentally obtained RSD, which corresponds to the intermediate precision here.

$$HorRat=\frac{RSD}{2\times c^{-0.15}}$$

The method was considered precise for each peptide with a HorRat below 2.0.

### 2.13 Limits of Detection and Quantitation

To evaluate the limit of detection (LOD) and limit of quantitation (LOQ) for every sample type, one sample (*n* = 6) of each type was selected and spiked at the beginning of sample preparation, according to the samples as described above, with different amounts of IS1–IS9 (0.003 ng to 2500 ng). The last spike-in step, where the identification criteria of the IS were fulfilled consistently (same ratios of five transitions and same retention time window as IS in water) was selected as LOD. The following samples were selected based on the presence of only traces of analytes for spike-in experiments: B15, V07, and S01. Sample preparation and analysis were the same as described above. LOQ was calculated by multiplying the estimated LOD by a factor of 3.3 to achieve the widely used ratio between LOD and LOQ.^29^

### 2.14 Recovery

Recovery was determined by diluting the B08 sample with gluten-free grain sorghum extract in two different ratios (50% and 75%). B08 was selected for recovery determination since it showed a comparable product substance (for example, viscosity and color) to the sorghum extract. Due to the unavailability of comparable analyte-free matrices for all analyzed samples, recovery was only determined for BME. Sample preparation of B08 was performed in triplicate (*n* = 3) for each dilution step as described previously for the absolute quantitation of CeD-active peptides in BME. Recovery was determined by comparing the analyzed CeD-active peptide concentration in diluted samples and the expected concentration calculated from the undiluted sample.^25^

### 2.15 Estimation of Gluten Content from the Peptide Concentrations

To estimate the gluten protein content, the complete sequences of the leading razor proteins for each peptide P1–P9 were used based on the identification of CeD-active peptides in the analyzed samples. A specific conversion factor for each peptide (*M*_Protein_/*M*_Peptide_) was calculated using the average molecular mass of the protein without the signal peptide, which was taken from the UniProtKB^23^ for the corresponding protein (Table 2). The total gluten protein content was calculated by multiplying the peptide concentrations by the respective factor and summing up all eight estimated gluten protein concentrations.

### 2.16 Statistics

Mean values and standard deviations of triplicates were calculated with Microsoft Office Excel 2016. One-way ANOVA with Tukey’s test (*p* ≤ 0.05) and Pearson correlations with *z*-score normalization of concentrations within a sample group were performed using OriginPro 2021b (OriginLab, Northampton, MA, USA). Normal distribution of the data was assessed using the Shapiro–Wilk test. The corresponding LOD was used for nondetectable CeD-active peptides, and a z-score normalization of the concentrations of every peptide within a sample group was performed before correlation. The following definition was used for Pearson correlation coefficients (*r*): ±0.54 < *r* ≤ ± 0.67: weak correlation; ±0.67 < *r* ≤ ± 0.78: medium correlation; ±0.78 < *r* ≤ ± 1.00: strong correlation.^30^

## 3 Results and Discussion

### 3.1 Identification of Gluten Peptides with CeD-Active Epitopes

The total number of identified gluten peptides varied for the different BMEs from nine to 162 per sample (Table 1). The lowest number of peptides was identified in B13 to B15, where the gluten content tested with R5c ELISA was also below the threshold of 20 mg/kg. For the remaining BMEs, B01 to B12, the R5c gluten content ranged from 2795.8 to 5416.6 mg/kg. In BMVs, the range of identified gluten peptides was from 13 to 111, with a gluten content below 20 mg/kg, except for V06, with 25.5 mg/kg. Two to 52 gluten peptides were identified in soy sauce, while the gluten content was under the LOQ of 10 mg/kg for all samples.

Among the identified peptides, some contained epitopes recognized by the R5 monoclonal antibody. In all BMEs besides B13, at least one peptide with R5 epitope was detected. For B01 to B12, the number ranged from 11 to 26 peptides, with B02 showing the highest number of peptides with R5 epitope. No R5 epitope was detected in V07, while the other six BMVs contained two to eight peptides with R5 epitope. In soy sauce, only one to 13 peptides with the R5 epitope were identified in six samples, and in nine soy sauces, no peptides that carried an R5 epitope were present.

The identified gluten peptide sequences were also filtered for CeD-active epitopes. Only peptides that contained an exact match of a complete 9-mer CeD-active epitope were considered.^4,31^ One to 21 peptides with at least one CeD-active epitope were identified in 14 BME, with B06 showing the highest number. Only B14 did not contain a single CeD-active peptide. One to five CeD-active peptides were identified in five BMVs, with V02 and V07 not containing any detectable CeD-active peptides. In ten soy sauces, one to seven CeD-active peptides were identified. Peptides with complete CeD-active epitopes were absent in the remaining five soy sauces.

It should be noted that the number of detected CeD-active peptides does not necessarily reflect the full spectrum of immunogenic sequences present in the samples. The analytical workflow relies on chymotryptic digestion, which means that only peptides containing at least one chymotrypsin cleavage site can be identified. Peptides that are already present in the food matrix as a result of fermentation or other processing steps, and that do not conform to chymotrypsin specificity, may remain undetected. Strategies that analyze both native peptides and peptides generated by chymotrypsin can provide a more comprehensive approach to gluten degradation during food processing. However, the enzymatic cleavage presented here led to reproducible detection of CeD-active peptides across different sample types.

A previous study reported the presence of gluten peptides in barley malt using various ELISA methods, with differences in antibody reactivity explained by different hydrolysis conditions during food processing.^12^ They found gluten contents between close to zero and 55,000 mg/L, depending on the sample and antibody used. In our study, similar variability was observed among the different BMEs. For example, no CeD-active peptides were detected in sample B14, whereas B06 contained 21 CeD-active peptides. Notably, B06 and B07 originated from the same manufacturer and shared the same best-before date but differed in color, which suggests identical starting material and procedures, with variation likely introduced during final drying.^32^ A higher drying temperature results in darker malts with altered flavor and peptide profile, as seen in the darker B07 sample compared to B06. While B07 exhibited a higher number of gluten peptides, it contained fewer CeD-active peptides than B06, suggesting that processing conditions significantly influence peptide composition and immunogenic potential.

Previous studies showed that components in soy sauce such as sugar can interfere with antibody-based immunoassays, which might potentially cause nonspecific inhibitory effects.^11,17,33^ This is consistent with our findings, where no gluten was detectable in soy sauce using R5c ELISA. However, Panda et al. (2017) noted that immunoreactive peptides might remain in soy sauces even after fermentation.^11^ Li et al. (2018) further investigated this using LC–MS/MS but did not detect any gluten peptides.^17^ In contrast, our results clearly demonstrate the presence of gluten peptides in soy sauces, including sequences carrying CeD-active epitopes. This discrepancy may be explained by the analytical platform used, because we used a nanoLC-system coupled to a high-resolution Orbitrap mass analyzer, which offers enhanced detection sensitivity and precision compared to high flow LC with lower-resolution MS systems.^34–36^ This allowed us to detect low-abundant peptides, which earlier studies may have failed to detect. Nevertheless, absolute quantitation is essential for a more comprehensive assessment of the potential CeD immunogenicity.

### 3.2 Comparison of Peptides with CeD-Active Epitopes

Figure 1A shows all sequences of identified CeD-active peptides in BME, BMV, and soy sauce. The peptide sequences are sorted by the presence of CeD-active epitopes designated according to Sollid et al. (2020).^4^ Two peptides (PQQPIPQQPQPY and PQQPQPFPQQPIPQQPQPY) were identified across all analyzed sample types (Figure 1B). Those two peptides were additionally the most frequently identified CeD-active peptides in 20 samples. Seven peptides were shared between two sample types, with SQQPIPQQPQPY only present in BME and BMV, and six other peptides, like PQQPQQPFPQPQQPFPW, only present in BME and soy sauce. Soy sauce and BMV did not share any peptides that were not also present in BME. Table S1 shows the number of samples for each sample type that contained the corresponding CeD-active peptides. In total, 54 CeD-active peptides were identified, of which 45 were only identified in one sample type including 32 that were unique in BME, seven in BMV, and six in soy sauce. Figure 1C additionally shows the overlap of identified peptides with R5 epitopes and CeD-active epitopes. 74 peptides with R5 epitopes were identified, which would thus contribute to the gluten determination by R5c ELISA. Meanwhile, 18 CeD-active peptides without R5 epitope have been identified, which would be overlooked in the R5c ELISA despite their immunogenic potential. The identified peptides had lengths between nine and 31 amino acids. Some peptides only contained one epitope sequence, all HLA-DQ2.5 restricted. The hordein-derived hor-3a epitope PIPQQPQPY occurred in 13 peptides. Some peptides contained epitope sequences originally designated as glia-epitopes according to Sollid et al. (2020),^4^ even though the corresponding samples were barley-based. For example, the glia-γ5 epitope occurred in eight peptide sequences identified in BME or BMV. This terminology reflects the historical identification of these epitopes in wheat gliadins rather than an exclusive taxonomic origin. Barley hordeins and wheat gliadins share extensive sequence homology, and many immunogenic motifs are conserved across species,^37^ and immunogenicity studies have mainly focused on wheat gluten.^4^ This is also evidenced by the shared sequences in wheat-based soy sauce and the other barley-based products, BME and BMV.^37^ The high sequence similarity is further illustrated by the occurrence of identical epitope sequences in both wheat- and barley-derived proteins. For example, glia-ω1 and hor-1 share the exact same epitope sequence,^38^ which were present in three of the shared sequences between soy sauce and BME. These findings are therefore not indicative of misidentification but reflect true biological homology between cereal gluten proteins, supported by high identification scores and consistent detection across technical replicates.

**Figure 1:**
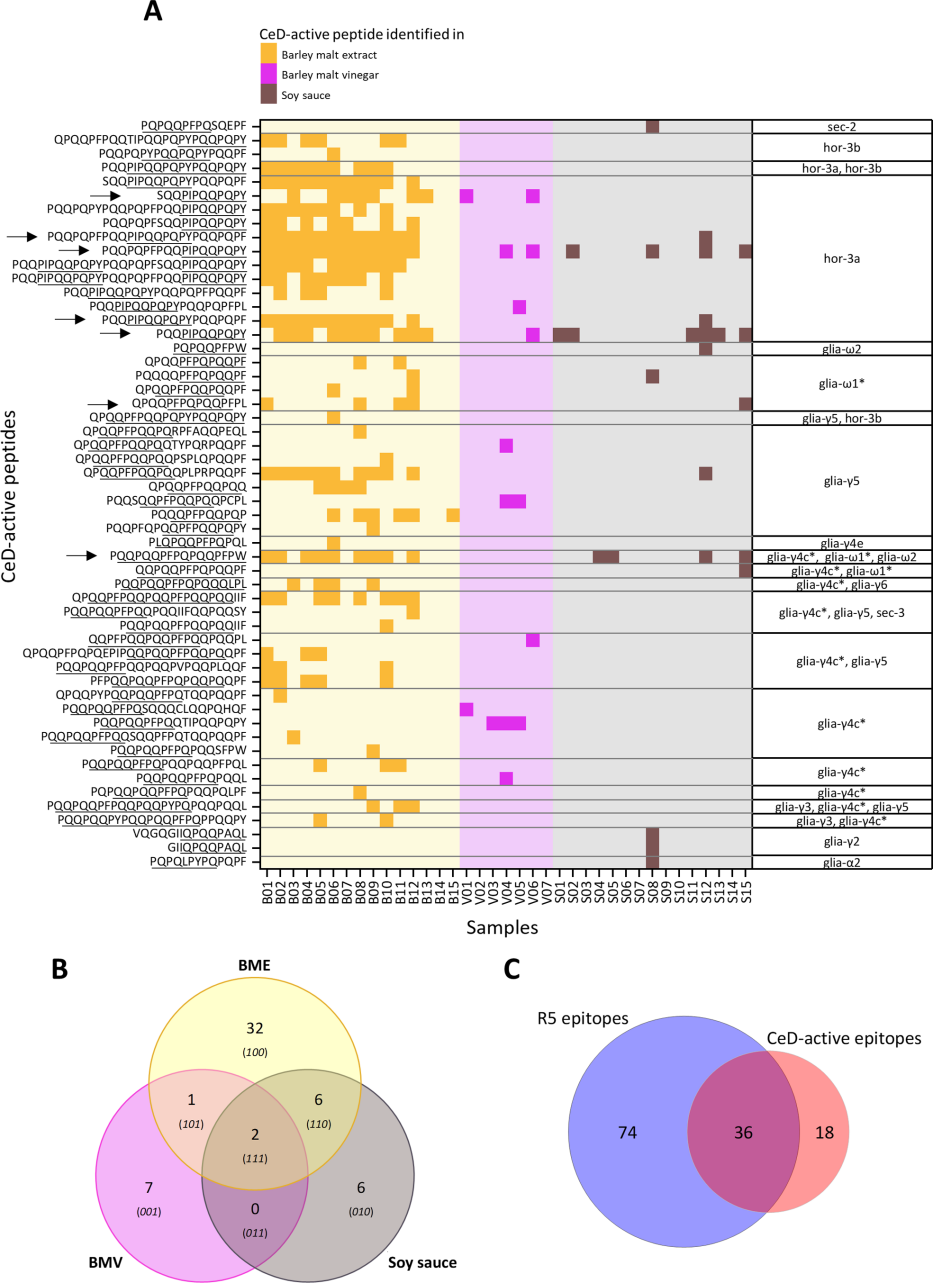
Identification of 54 peptides with at least one celiac disease (CeD)-active epitope in barley malt extract (B01 to B15), barley malt vinegar (V01 to V07), and soy sauce (S01 to S15) based on untargeted nanoliquid chromatography-tandem mass spectrometry (nanoLC-MS/MS) analysis. (A) Heatmap of identified CeD-active peptides in samples, epitope sequences according to Sollid et al. (2020) are underlined and listed in the table on the right side of the heatmap. Epitopes marked with an * share identical sequences after reversion of deamidation (glia-ω1, hor-1 and sec-1; glia-γ4c and glia-γ1a). Arrows mark peptides that were selected for the absolute quantitation. (B) Numbers of identified CeD-active peptides that are unique or common between the analyzed samples: barley malt extract (BME, *n* = 15), barley malt vinegar (BMV, *n* = 7) and soy sauce (*n* = 15). (C) Numbers of identified peptides with at least one R5 epitope or CeD-active epitope and their overlap.

The heatmap provides an overview of the peptides frequently detected across multiple samples, including those shared between different sample types and peptides unique to a specific sample type. For the purpose of identifying robust and stable marker peptides, modifications besides oxidation, acetylation, and carbamidomethylation were not included for peptide identification, although food processing can produce peptides with post-translational modifications.^39^ Including such modifications could lead to a more comprehensive identification of possible peptides present in foods, but it would have introduced additional variability and complexity into the interpretation of peptide abundances, particularly in the context of absolute quantitation. Furthermore, although the database used for peptide identification was restricted to gluten-related protein sequences, this limitation did not comprise the objective of this study, which focused exclusively on detecting gluten-derived and CeD-active peptides. However, the absence of a soy protein database means that the broader proteome of soy sauce was not assessed, and the possibility of missing unrelated peptides cannot be excluded. Nevertheless, the identified sequences in soy sauce showed high identification scores, consistent fragmentation patterns, and reproducible identification across replicates. Several peptides detected in soy sauce were also found in BME and BMV (Figure 1B), supporting the validity of their identification despite matrix complexity and without incorporating all possibly present peptides within a sample. Therefore, the identification of CeD-active peptides in this study enabled the selection of peptides for absolute quantitation, which provides more information for further assessment of the present CeD-active peptides in those samples.

### 3.3 Development of the Targeted NanoLC-MS/MS Method

The CeD-active peptides for absolute quantitation with targeted nanoLC-MS/MS were selected based on the identified CeD-active peptides in the samples using untargeted nanoLC-MS/MS. Many of these CeD-active peptides had already been analyzed in our previous study of gluten-free barley beers and corresponded to peptides P1 to P7.^40^ Furthermore, CeD-active peptides that were exclusively identified in the present study were added to the selection (P8 and P9). Eleven peptides were initially preselected and signal quality as well as the availability of at least five transitions at the same retention time were evaluated in targeted pre-experiments. Peptide identification was verified by measuring full scan spectra with MS2 of the samples and comparing the retention time and precursor to product ion transitions with those of the synthesized peptides. The exclusion of some peptides due to insufficient signal quality and absence in many samples resulted in a final selection of eight peptides for SIDA (Table 2). Further, the 2+ charge state was selected as precursor ion with the highest intensity for P1, P5, P6, P7, P8, and P9, while the 3+ charge state showed higher intensities for P2 and P3. Five precursor to product ion transitions were selected (Table 2) and the MS/MS settings optimized experimentally to achieve the highest signal intensity with at least ten scan events per peak.

### 3.4 Method Validation

Precision, LOD, LOQ and recovery of the SIDA method for quantitating CeD-active peptides in food products with partially hydrolyzed gluten were determined (Table 3). Repeatability was very good across all peptides and samples, ranging from 2.2% to 9.7%. Intermediate precision was also very good for BME and soy sauce (2.1–7.0%), whereas it was higher for BMV (13.3–21.8%), due to the low peptide concentrations of CeD-active peptides in BMV. The HorRat values were between 0.1 and 0.9, indicating acceptable precision. However, the low HorRat values for CeD-active peptides in BMV and soy sauce can be attributed to the low concentration range.

**Table 3:** Repeatability (*n* = 6), Intermediate Precision (*n* = 18), Horwitz Ratio (HorRat), Limit of Detection (LOD), Limit of Quantitation (LOQ), and Recovery of the different Celiac Disease-Active Peptides in Barley Malt Extract (BME), Barley Malt Vinegar (BMV) and Soy Sauce, Analyzed by Targeted Nano-Liquid Chromatography-Tandem Mass Spectrometry with Stable Isotope Dilution Assay^a^

			P1	P2	P3	P5	P6	P7	P8	P9
BME	precision	repeatability [ %]	3.4	6.6	5.5	3.0	9.7	4.7	2.6	4.52
		intermediate [ %]	4.6	5.2	7.0	2.8	5.2	2.9	4.0	6.1
		HorRat	0.6	0.8	0.9	0.2	0.6	0.2	0.5	0.5
	sensitivity	LOD [mg/kg]	4.1 × 10^–5^	0.4	40.5	0.04	3.8	0.20	0.008	0.04
		LOQ [mg/kg]	1.4 × 10^–4^	1.3	133.7	0.1	12.4	0.7	0.03	0.1
	recovery	in 50% BME [ %]	101.1	110.9	79.1	94.6	102.9	99.7	94.1	102.3
		in 75% BME [ %]	96.5	98.5	82.1	93.6	91.3	104.4	90.2	97.7
BMV	precision	repeatability [ %]	9.0	5.4			3.8		2.9	
		intermediate [ %]	16.0	21.8			14.3		13.3	
		HorRat	0.4	0.6			0.4		0.6	
	sensitivity	LOD [mg/kg]	5.0 × 10^–6^	5.0 × 10^–6^	2.5 × 10^–4^	5.0 × 10^–5^	9.2 × 10^–5^	4.9 × 10^–5^	5.0 × 10^–6^	4.6 × 10^–5^
		LOQ [mg/kg]	1.7 × 10^–5^	1.7 × 10^–5^	8.2 × 10^–4^	1.7 × 10^–4^	3.0 × 10^–4^	1.6 × 10^–4^	1.7 × 10^–5^	1.5 × 10^–4^
soy sauce	precision	repeatability [ %]	2.8	2.2						
		intermediate [ %]	4.2	2.1						
		HorRat	0.2	0.1						
	sensitivity	LOD [mg/kg]	8.8 × 10^–6^	0.04	2.2	0.004	0.04	0.004	8.7 × 10^–4^	0.004
		LOQ [mg/kg]	2.9 × 10^–5^	0.1	7.2	0.01	0.1	0.01	0.003	0.01

a Precision was only calculated for the analytes that were detected in those samples.

Accurate quantitation of CeD-active peptides in food samples requires a highly sensitive method to detect even trace amounts of partially hydrolyzed gluten. The method showed high sensitivity for most CeD-active peptides in BME, with LODs ranging from 4.1 × 10^–5^ mg/kg to 3.8 mg/kg and LOQs from 1.4 × 10^–4^ mg/kg to 12.4 mg/kg. Peptide P3 showed a higher LOD of 40.5 mg/kg and a higher LOQ of 133.7 mg/kg, which is consistent with results in BMV and soy sauce, where P3 also showed the highest limits compared to the other peptides (Table 3). The LODs of the remaining peptides showed high sensitivity, ranging from 5.0 × 10^–6^ mg/kg to 2.5 × 10^–4^ mg/kg in BMV and 8.8 × 10^–6^ mg/kg to 0.04 mg/kg in soy sauce. The LOQs varied from 1.7 × 10^–5^ mg/kg to 8.2 × 10^–4^ mg/kg in BMV and from 2.9 × 10^–5^ mg/kg to 0.1 mg/kg in soy sauce.

The method also demonstrated very good recoveries between 79.1% and 110.9% for 50% diluted BME and between 82.1% and 104.4% for 75% diluted BME. These results are within the commonly accepted range of 70% to 120%,^41^ confirm the reliability of the developed SIDA method, and indicate that peptide extraction efficiency remains consistent in both undiluted and sorghum malt-diluted BME samples. The observed differences in LOD and LOQ across sample types highlight the strong influence of matrix composition on analytical accuracy. This emphasizes the importance of matrix-specific method validation with SIDA instead of external calibration in solvent.^42^ The LODs and precision of the CeD-active peptides in BME were comparable to those reported for amylase/trypsin-inhibitor analysis in wheat and barley using SIDA and LC–MS/MS, where LODs reached up to 3.9 mg/kg and precision values up to 15.6%.^25,43^

### 3.5 Absolute Quantitation of Celiac Disease-Active Peptides

Eight CeD-active peptides were quantitated with absolute peptide concentrations using SIDA and targeted nanoLC-MS/MS across 15 BME, seven BMV, and 15 soy sauce samples (Table 4). B01 to B12 had total concentrations of CeD-active peptides ranging from 481.1 mg/kg to 1304.7 mg/kg (Figure 2A). B13 to B15 contained only very low levels of CeD-active peptides near the LOQ, with the total peptide concentrations ranging from 0.06 mg/kg to 0.1 mg/kg. All peptides were quantitated in B01 to B12, whereas in B13 to B15, only P1 and P8 showed a concentration above the LOQ. P2 exhibited the highest concentrations among all peptides in all BME samples, ranging from 210.6 mg/kg to 563.0 mg/kg, and the lowest concentrations were found for P6 and P8, with 7.3 mg/kg at most. Among the BMV, CeD-active peptides were present in concentrations from 0.011 mg/kg to 0.042 mg/kg in six of seven samples (Figure 2B), whereas all peptides were under the LOD in V07. P3 and P7 were not detectable in any BMV. V06, with the highest total peptide concentration, was the only BMV where six out of eight CeD-active peptides were above the LOQ. Only P1, P6, and P8 were quantitated in V01–V06, with P8 showing the highest concentrations (0.024 mg/kg in V01). In all 15 soy sauces, only P1 and P2 were detectable with concentrations above the LOQ (Figure 2C). The total peptide concentrations ranged from 0.20 to mg/kg 0.22 mg/kg for 13 soy sauces, and two deviating samples with a total peptide concentration of 0.52 mg/kg in S12 and 0.39 mg/kg in S13.

**Table 4:** Concentrations of Selected Celiac Disease-Active Peptides (P1–P9) in Food Products with Partially Hydrolyzed Gluten, Barley Malt Extract (B01–B15), Barley Malt Vinegar (V01–V07), and Soy Sauce (S01–S15) Analyzed by Targeted Nano-Liquid Chromatography-Tandem Mass Spectrometry with Stable Isotope Dilution Assay^a^ ^,^ ^b^

peptide concentration [mg/kg]										estimated total gluten content [mg/kg]
ID	P1	P2	P3	P5	P6	P7	P8	P9	Total	
barley malt extract										
B01	98.3 ± 1.7	492.4 ± 36.2	472.2 ± 61.9	7.1 ± 0.6	44.0 ± 2.2	3.8 ± 0.1	134.0 ± 8.9	3.6 ± 0.3	1255.4 ± 116.2	16,490
B02	111.9 ± 6.4	484.8 ± 24.4	426.6 ± 57.9	6.4 ± 0.1	41.0 ± 1.8	3.6 ± 0.3	152.3 ± 21.8	3.5 ± 0.3	1231.3 ± 114.6	16,242
B03	108.1 ± 8.3	535.6 ± 37.3	413.1 ± 36.5	5.3 ± 0.4	46.7 ± 5.0	2.5 ± 0.2	162.6 ± 20.8	2.7 ± 0.3	1279.6 ± 108.7	16,851
B04	82.2 ± 4.3	387.0 ± 21.7	305.0 ± 19.2	5.9 ± 0.2	33.0 ± 3.7	3.0 ± 0.2	104.8 ± 7.4	2.7 ± 0.2	923.5 ± 56.7	12,331
B05	66.3 ± 8.6	284.1 ± 29.7	222.0 ± 30.7	3.5 ± 0.5	24.3 ± 4.3	2.9 ± 0.3	65.4 ± 1.6	2.4 ± 0.4	671.0 ± 75.6	9115
B06	107.6 ± 3.4	563.0 ± 113.2	425.9 ± 25.7	6.4 ± 0.08	42.1 ± 0.7	3.0 ± 0.2	152.5 ± 23.2	4.3 ± 0.3	1304.7 ± 166.4	17,320
B07	93.8 ± 5.5	473.1 ± 37.4	374.5 ± 25.1	7.4 ± 0.5	37.3 ± 3.2	4.4 ± 0.3	122.3 ± 10.6	3.9 ± 0.2	1116.7 ± 82.7	14,886
B08	102.2 ± 2.7	498.5 ± 57.5	490.4 ± 18.6	9.2 ± 0.7	41.3 ± 2.4	4.7 ± 0.4	139.9 ± 2.9	3.1 ± 0.1	1289.4 ± 85.2	16,907
B09	99.9 ± 5.2	512.7 ± 47.9	385.1 ± 60.6	10.5 ± 2.0	43.7 ± 9.9	5.7 ± 1.5	118.4 ± 14.6	3.0 ± 0.3	1178.9 ± 141.7	15,868
B10	81.5 ± 5.8	416.2 ± 26.6	338.9 ± 45.2	7.8 ± 1.7	35.0 ± 4.6	4.7 ± 0.8	101.8 ± 5.1	3.5 ± 0.5	989.4 ± 89.9	13,213
B11	51.0 ± 4.0	210.6 ± 12.8	177.2 ± 11.3	6.5 ± 0.8	14.3 ± 0.6	4.9 ± 0.6	12.7 ± 2.5	4.0 ± 0.1	481.1 ± 32.5	6839
B12	67.4 ± 1.8	305.0 ± 26.5	331.7 ± 11.0	11.3 ± 1.5	28.3 ± 4.1	7.3 ± 0.8	163.6 ± 22.0	6.2 ± 0.4	920.9 ± 67.6	11,554
B13	0.03 ± 0.004	<LOQ	<LOQ	<LOD	<LOD	<LOD	0.1 ± 0.02	<LOD	0.1 ± 0.02	1.28
B14	0.03 ± 0.0001	<LOD	<LOD	<LOD	<LOD	<LOD	0.05 ± 0.002	<LOD	0.08 ± 0.002	0.99
B15	0.03 ± 0.002	<LOD	<LOD	<LOD	<LOD	<LOD	0.03 ± 0.001	<LOD	0.06 ± 0.003	0.88
barley malt vinegar										
V01	10.6 ± 2.5 × 10^–4^	<LOD	<LOD	<LOQ	15.8 ± 1.0 × 10^–4^	<LOD	0.024 ± 0.0059	<LOD	0.027 ± 0.0062	0.19
V02	5.8 ± 0.1 × 10^–4^	<LOD	<LOD	<LOD	7.3 ± 0.5 × 10^–4^	<LOD	0.012 ± 0.0015	<LOD	0.014 ± 0.0016	0.095
V03	5.9 ± 1.7 × 10^–4^	<LOD	<LOD	<LOD	12.1 ± 1.2 × 10^–4^	<LOD	0.018 ± 0.0009	<LOD	0.020 ± 0.0012	0.14
V04	10.1 ± 1.1 × 10^–4^	<LOD	<LOD	<LOD	19.0 ± 3.4 × 10^–4^	<LOD	0.020 ± 0.0029	<LOD	0.023 ± 0.0034	0.17
V05	3.9 ± 0.6 × 10^–4^	<LOD	<LOD	<LOD	8.8 ± 0.2 × 10^–4^	<LOD	0.010 ± 0.0007	<LOD	0.011 ± 0.0008	0.079
V06	31.0 ± 7.7 × 10^–4^	0.011 ± 0.0012	<LOD	8.2 ± 1.0 × 10^–4^	0.0079 ± 0.0015	<LOD	0.019 ± 0.0003	3.1 ± 0.1 × 10^–4^	0.042 ± 0.0070	0.48
V07	<LOD	<LOD	<LOD	<LOD	<LOD	<LOD	<LOD	<LOD	<LOD	
Soy sauce										
S01	0.041 ± 0.0015	0.17 ± 0.0029	<LOD	<LOD	<LOD	<LOD	<LOD	<LOD	0.21 ± 0.0044	3.49
S02	0.045 ± 0.0017	0.17 ± 0.0028	<LOD	<LOD	<LOD	<LOD	<LOD	<LOD	0.21 ± 0.0045	3.56
S03	0.043 ± 0.0035	0.17 ± 0.0035	<LOD	<LOD	<LOD	<LOD	<LOD	<LOD	0.22 ± 0.0070	3.56
S04	0.040 ± 0.0008	0.17 ± 0.0030	<LOD	<LOD	<LOD	<LOD	<LOD	<LOD	0.21 ± 0.0038	3.46
S05	0.039 ± 0.0007	0.17 ± 0.0003	<LOD	<LOD	<LOD	<LOD	<LOD	<LOD	0.21 ± 0.0010	3.43
S06	0.041 ± 0.0035	0.17 ± 0.0044	<LOD	<LOD	<LOD	<LOD	<LOD	<LOD	0.21 ± 0.0078	3.56
S07	0.041 ± 0.0047	0.17 ± 0.0061	<LOD	<LOD	<LOD	<LOD	<LOD	<LOD	0.21 ± 0.011	3.50
S08	0.040 ± 0.0014	0.17 ± 0.0068	<LOD	<LOD	<LOD	<LOD	<LOD	<LOD	0.21 ± 0.0082	3.42
S09	0.042 ± 0.0021	0.16 ± 0.0042	<LOD	<LOD	<LOD	<LOD	<LOD	<LOD	0.22 ± 0.0063	3.58
S10	0.040 ± 0.0029	0.17 ± 0.0045	<LOD	<LOD	<LOD	<LOD	<LOD	<LOD	0.20 ± 0.0074	3.37
S11	0.039 ± 0.0013	0.2 ± 0.0051	<LOD	<LOD	<LOD	<LOD	<LOD	<LOD	0.21 ± 0.0063	3.51
S12	0.10 ± 0.014	0.42 ± 0.015	<LOD	<LOD	<LOD	<LOD	<LOD	<LOD	0.52 ± 0.030	8.66
S13	0.084 ± 0.0071	0.30 ± 0.017	<LOD	<LOD	<LOD	<LOD	<LOD	<LOD	0.39 ± 0.024	6.48
S14	0.041 ± 0.0018	0.16 ± 0.0012	<LOD	<LOD	<LOD	<LOD	<LOD	<LOD	0.20 ± 0.0030	3.35
S15	0.040 ± 0.0030	0.16 ± 0.0030	<LOD	<LOD	<LOD	<LOD	<LOD	<LOD	0.20 ± 0.0060	3.37

a LOD: limit of detection; LOQ: limit of quantitation.

b Total peptide concentration is the summed concentration of all eight peptides in one sample. The estimated total gluten content is calculated from the peptide concentrations multiplied by the corresponding conversion factors (Table 2) and summed up.

**Figure 2:**
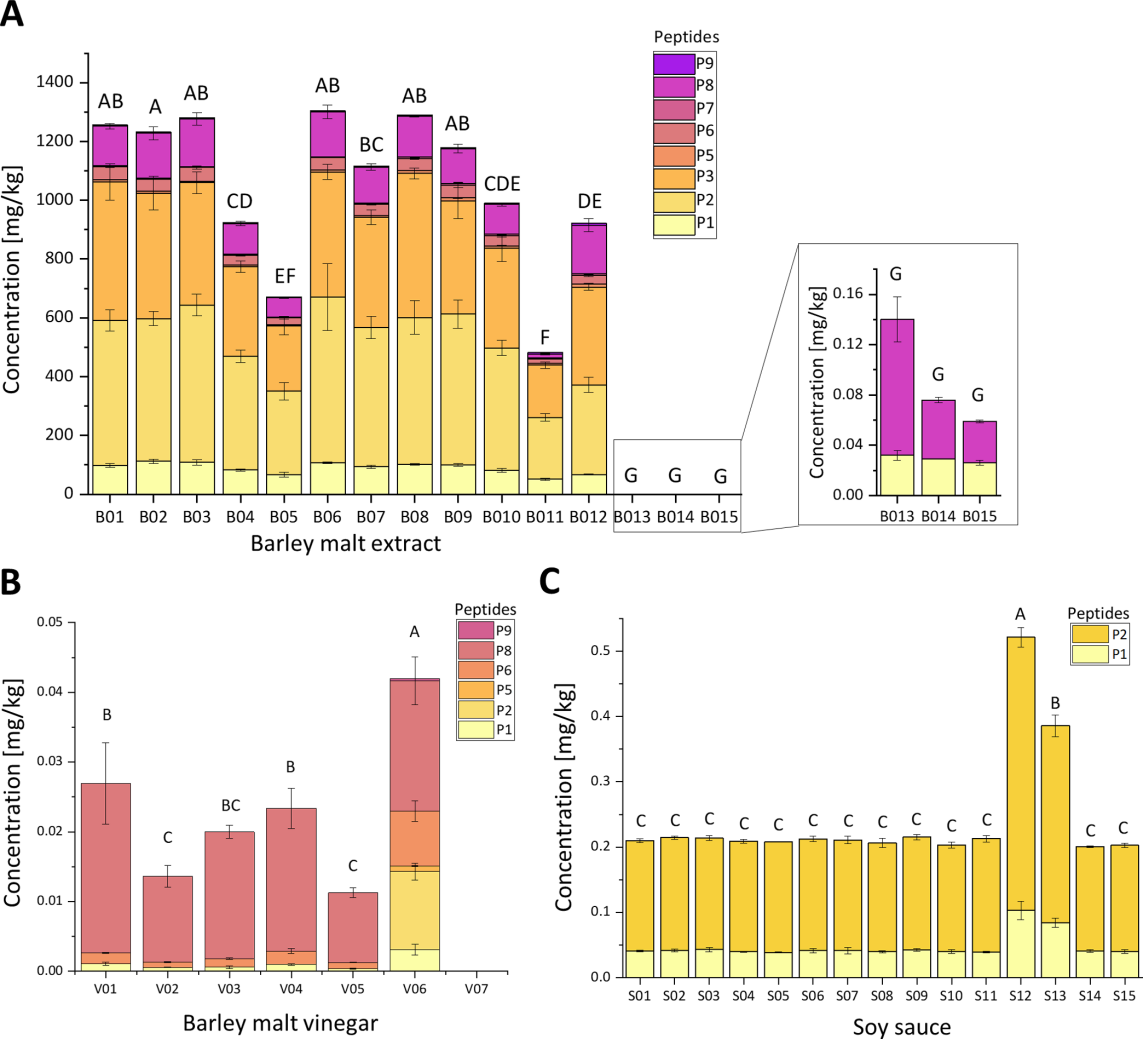
Concentrations of eight celiac disease-active peptides in (A) barley malt extract (B01 to B15), (B) barley malt vinegar (V01 to V07), and (C) soy sauce (S01 to S15), analyzed by targeted nanoliquid chromatography-tandem mass spectrometry with stable isotope dilution assay. Different capital letters indicate significant differences between the samples for the total concentration (one-way ANOVA with Tukey’s test, *p* ≤ 0.05).

Overall, P1 was the most frequently detected peptide across all analyzed samples, with signals observed in 36 out of 37 samples. In contrast, P3 and P7 were only quantitated above the LOQ in 12 BMEs (B01 to B12), while P5 and P9 were detected in those 12 BMEs and in one BMV (V06). The use of chymotrypsin during sample preparation has limitations due to its less specific cleavage compared to trypsin, which can lead to variable peptide generation.^44^ Nevertheless, chymotrypsin remains the preferred enzyme for the analysis of CeD-active peptides^16^ and demonstrated acceptable reproducibility across different partially hydrolyzed and fermented food matrices (Table 3). This is further supported by the relative peptide composition observed in the analyzed samples (Figure S1). For BMEs B01 to B12 and BMVs V01 to V05, comparable peptide profiles were found. In contrast, samples B13 to B15 and V06 showed different relative compositions, because fewer peptides were detected. Interestingly, despite significant differences in the total concentration of CeD-active peptides in soy sauces S12 and S13, the relative peptide composition remained consistent with the other samples.

Together with the absolute quantitation of CeD-active peptides, this study provides a comprehensive insight into the peptide profiles of various partially hydrolyzed and fermented foods. This enables an evaluation of the degree of hydrolysis within food products, even when using a limited predefined set of peptides for SIDA.

Surprisingly, S01, a wheat-free soy sauce, had a total peptide concentration of 0.21 mg/kg. Panda and Garber (2019) previously showed the presence of a protein band in a gluten-free soy sauce using Western blot, which they explained through possible gluten contamination or incomplete proteolysis during fermentation.^45^ In our study, the presence of the CeD-active gluten peptides was clearly confirmed in S01 (Table 1, Table 4), supporting the findings by Panda and Garber (2019).^45^

Our study is the first to report absolute quantities of CeD-active peptides in BMVs. Li et al. (2018) had previously identified gluten peptides with CeD-active epitopes in malt vinegars, including the epitopes glia-ω1a and glia-γ4c.^17^ However, our approach enabled not only identification but also the absolute quantitation of CeD-active peptides. This quantitative knowledge of remaining CeD-active peptides and the peptide composition in food products with partially hydrolyzed gluten can serve as a foundation for further evaluation of their immunogenic potential, which is beyond the scope of the present work. Furthermore, this analytical approach offers a valuable tool for monitoring the efficiency of gluten-reducing processes by focusing on peptides that pose a potential risk to CeD patients. By analyzing the barley malt extract used for the production of other foods, each processing step can be evaluated with regard to the peptide degradation and reduction during the process, allowing for a comparative assessment of gluten-reducing strategies.

### 3.6 Comparison of Quantities of CeD-Active Peptides and Gluten Content

Pearson correlations were calculated for the concentrations of the single CeD-active peptides, the total concentration of CeD-active peptides, the gluten content measured by R5c ELISA, and the crude protein content of the analyzed BMEs (Figure 3) and BMVs (Figure S2A). For soy sauce (Figure S2B), the R5c gluten content was excluded from the correlation analysis, because all results were below the LOQ (Table 1). In BMEs, all single CeD-active peptides showed strong correlations with the total concentrations of CeD-active peptides. Especially, P1, P2, P3, and P6 showed strong correlations with *r* = 0.99. The concentrations of individual peptides were also strongly correlated with the R5c ELISA gluten content, ranging from *r* = 0.82 (P7) to *r* = 0.91 (P9). The total concentration of CeD-active peptides and R5c ELISA gluten content also showed a strong correlation (*r* = 0.90). Weak to strong correlations were obtained between individual peptide concentrations and protein content, with P7 showing the weakest coefficient (*r* = 0.60) and P1 showing the highest coefficient (*r* = 0.83). Both total peptide concentration (*r* = 0.79) and gluten content (*r* = 0.82) also correlated strongly with protein content. For BMV, similar correlations were obtained. The concentrations of all single CeD-active peptides detected in BMV were correlated with the total peptide concentrations with *r* = 0.74–0.92 (Figure S2A). The total concentration of the CeD-active peptides was also strongly correlated with the gluten content (*r* = 0.91). Besides P8, each individual CeD-active peptide showed a strong correlation with the gluten content, with a coefficient up to 0.98 for P1. In contrast to BME, no correlations were observed between the protein content and most individual peptide concentrations, total summed peptide concentrations and the gluten content. In soy sauce (Figure S2B), the concentration of the individual peptides even showed a weak negative correlation to the protein content (*r* = −0.66).

**Figure 3:**
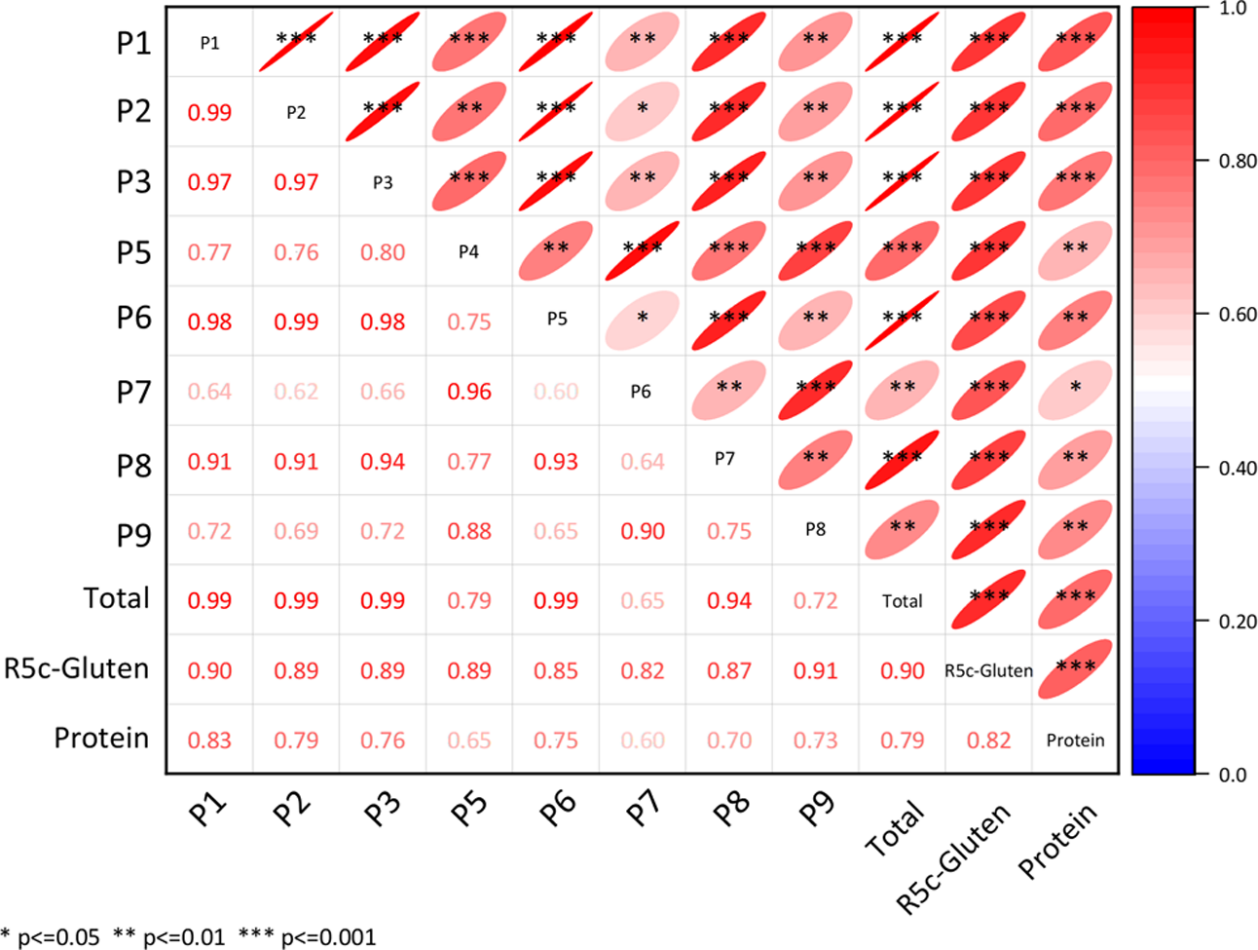
Correlation matrix of the absolute concentration of celiac disease-active peptides P1 to P9, the summed total concentration of the peptides, the gluten content and crude protein content in barley malt extract (*n* = 15). Analyses were performed with R5 competitive enzyme-linked immunosorbent assay (R5c ELISA) for R5c-gluten content, Dumas combustion method for protein content and nanoliquid chromatography-tandem mass spectrometry with stable isotope dilution assay for the concentration of peptides P1–P9.

Together, these results demonstrate that a direct correlation between crude protein content and concentration of CeD-active peptides cannot be assumed in fermented and partially hydrolyzed food products. In BME, strong correlations between peptide concentration, gluten content, and protein content were observed, similar to correlations seen for intact gluten in wheat flour.^30^ However, this relationship no longer applies to more extensively hydrolyzed products, such as BMV and soy sauce. This is plausible because the protein content in such products, determined via nitrogen content, is additionally obtained from free amino acids and other nitrogenous compounds formed during processing.^46^ The lower peptide concentration observed in BMV further supports the lack of correlation between protein and gluten content.

As suggested by Panda and Garber (2019), these findings can help to recognize differences in the proteolytic patterns among different production processes. Additionally, the knowledge of absolute peptide quantities could be valuable for future in vivo and in vitro immunotoxicity assays of such peptides.^45^

However, the direct determination of total gluten content using current MS techniques still remains challenging. The peptide concentration cannot be directly converted into gluten content due to the heterogeneous nature of gluten proteins and the variable modifications they undergo during food processing.^47^ Nevertheless, considering that the legal threshold for gluten is defined as 20 mg of gluten proteins per kg of the product, this study performed a conceptual estimation of gluten content based on the concentrations of CeD-active peptides (Table 4), in order to classify it within the regulatory framework. An intended overestimation resulted by estimating the protein amounts for every single analyzed CeD-active peptide, even if they might originate from the same protein. This provides insights into a rather conservative upper-bound estimate (worst-case scenario), which allows an initial indication of whether the residual partially hydrolyzed gluten amounts fall far below, near, or above the regulatory threshold. Similar considerations were described recently for gluten-free beers, where the peptide-based calculations were shown to provide an approximation of the order of magnitude rather than exact measurement of gluten levels.^40^ Based on this estimation, the gluten protein content for BMEs B01 to B12 ranged from 6839 and 17,320 mg/kg. This was expected, as BME is not intended to meet the gluten-free criteria. Nevertheless, such values become more relevant when products incorporate small quantities of BME for flavor adjustments. In BMVs V01 to V06, the estimated gluten protein content ranged from 0.095 mg/kg to 0.48 mg/kg, and in the 15 soy sauces, from 3.35 mg/kg to 8.66 mg/kg, both clearly below the 20 mg/kg threshold. In contrast, the R5c ELISA results indicated higher gluten contents for BMVs compared to soy sauce, ranging from 13.2 mg/kg to 25.5 mg/kg (Table 1). Our study identified more peptides containing the R5 epitopes QQPFP, QQQFP, LQPFP, and QLPFP^8^ than CeD-active epitopes in BMVs (Table 1), which may explain the elevated gluten values for BMV obtained by R5c ELISA. For example, in V04, eight peptides with the R5 epitope were identified, while only five peptides contained a CeD-active epitope. Notably, previous work has shown that commercially available gluten-free barley beers contained potentially CeD-active peptides that would be overlooked in cELISA analysis and therefore pose a potential risk to individuals with CeD.^10^ The present study found a significant correlation between the gluten content measured by R5c ELISA and the total concentration of CeD-active peptides in BMVs. However, major discrepancies were observed when comparing the results of BMVs and soy sauces. Although soy sauces contained a total concentration of CeD-active peptides at least five times higher than in BMVs (Table 4), their R5c gluten content was below the LOQ and thus below that of BMVs. This leads to the assumption that certain food matrices might interfere more with current analytical detection methods, likely due to higher LODs, LOQs, and matrix-specific effects.

Since the currently used reference materials do not adequately represent the different hydrolytic conditions across different food types,^12^ immunoassay-based methods alone may fail to accurately determine the gluten content. This underscores the value of combining ELISA, which is a comparably rapid and accessible method, with LC–MS/MS for a more detailed and accurate assessment of the peptide profile in those samples. Such an approach enables the identification and quantitation of potentially immunogenic peptides, even after extensive protein modification during processing. For now, it still remains a problem to relate the quantities obtained from either ELISAs or proteomics to the immunogenic potential of these samples. Bridging this gap will require some in vivo toxicity assessment to determine whether trace amounts of CeD-active peptides can elicit adverse clinical responses in individuals with CeD.

This study demonstrates that foods containing partially hydrolyzed gluten, such as BME, BMV, and soy sauces, can still contain CeD-active peptides, even when classified as gluten-free by immunoassays like R5c ELISA. Using a sensitive nanoLC-MS/MS approach combined with SIDA, we identified and quantitated eight CeD-active peptides across a range of partially hydrolyzed and fermented food matrices. This revealed significant differences in peptide concentrations, which were not always reflected in the gluten content measured by ELISA, particularly in highly fermented matrices like soy sauce. The combination of ELISA for rapid screening and LC–MS/MS for detailed peptide profiling offers a more accurate assessment of gluten content and potential immunogenicity of remaining CeD-active peptides. Moreover, the identification of CeD-active marker peptides and their absolute quantities provide a valuable basis for further in vitro and in vivo immunotoxicity studies, as well as for evaluating the effectiveness of gluten-reducing processing strategies. Finally, this work highlights the need for improved reference materials and analytical methods that account for the complexity of partially hydrolyzed and fermented food matrices. Until the immunogenic potential of trace peptide levels can be reliably linked to clinical effects, a comprehensive risk assessment of such products remains essential to ensure the safety of individuals with CeD.

## Supplementary Material



## Data Availability

The mass spectrometry untargeted proteomics data have been deposited to the ProteomeXchange Consortium via the PRIDE (Perez-Riverol et al., 2022) partner repository with the data set identifier PXD068276. Targeted mass spectrometry data are publicly available on Panorama Public (https://panoramaweb.org/Og5t79.url).

## Notes

Overview of peptides with at least one celiac disease (CeD)-active epitope (Table S1), peptide proportions in the samples (Figure S1), correlation matrices for barley malt vinegar and soy sauce (Figure S2) and exemplary response line for peptide P1 (Figure S3) (PDF)
