# Supplementary material for: Proteomics Analysis of Celiac Disease-Active Peptides in Food Products with Partially Hydrolyzed Gluten
Source: J Agric Food Chem. 2026 Jan 12;74(3):3013–27. doi: 10.1021/acs.jafc.5c13719 (PMC12862762; doi:10.1021/acs.jafc.5c13719)
Supplement: Supplementary file 1 [file jf5c13719_si_001.pdf]

Supplementary Material

Proteomics Analysis of Celiac Disease-Active Peptides in Food Products with Partially Hydrolyzed Gluten

Eleonora Tissen[1,2], Lana Dakwar[1,2], Sabrina Geisslitz[2], Katharina Anne Scherf[2,1*]

[1] Technical University of Munich, TUM School of Life Sciences, Professorship of Food Biopolymer Systems, Lise-Meitner-Str. 34, 85354 Freising, Germany

[2] Leibniz Institute for Food Systems Biology at the Technical University of Munich, Lise-Meitner-Str. 34, 85354 Freising, Germany

Table S1: Identification of 54 peptides with at least one celiac disease (CeD)-active epitope in barley malt extract (*n* = 15), barley malt vinegar (*n* = 7) and soy sauce (*n* = 15) based on untargeted nano-liquid chromatography-tandem mass spectrometry analysis. The number shows in how many samples of each sample type the corresponding peptide was identified. Underlined sequences are R5 recognition epitopes of the R5 competitive enzyme-linked immunosorbent assay commonly used for gluten determination.

	Number of identifications in		
	**Barley malt extract**	**Barley malt vinegar**	**Soy sauce**
PQPQQPFPQSQEPF	0	0	1
QPQQPFPQQTIPQQPQPYPQQPQPY	6	0	0
PQQPQPYPQQPQPYPQQPF	1	0	0
PQQPIPQQPQPYPQQPQPY	9	0	0
SQQPIPQQPQPYPQQPQPF	11	0	0
SQQPIPQQPQPY	7	2	0
PQQPQPYPQQPQPFPQQPIPQQPQPY	8	0	0
PQQPQPFSQQPIPQQPQPY	7	0	0
PQQPQPFPQQPIPQQPQPYPQQPQPF	12	0	1
PQQPQPFPQQPIPQQPQPY	12	2	4
PQQPIPQQPQPYPQQPQPFSQQPIPQQPQPY	10	0	0
PQQPIPQQPQPYPQQPQPFPQQPIPQQPQPY	8	0	0
PQQPIPQQPQPYPQQPQPFPQQPF	4	0	0
PQQPIPQQPQPYPQQPQPFPL	0	1	0
PQQPIPQQPQPYPQQPQPF	11	0	1
PQQPIPQQPQPY	10	1	6
PQPQQPFPW	0	0	1
QPQQPFPQPQQPF	2	0	0
PQQQQPFPQPQQPF	1	0	1
QPQQPFPQQPQQPF	2	0	0
QPQQPFPQPQQPFPL	4	0	1
QPQQPFPQQPQPYPQQPQPY	1	0	0
QPQQPFPQQPQRPFAQQPEQL	1	0	0
QPQQPFPQQPQQTYPQRPQQPF	0	1	0
QPQQPFPQQPQQPSPLQPQQPF	1	0	0
QPQQPFPQQPQQPLPRPQQPF	10	0	1
QPQQPFPQQPQQ	4	0	0
PQQSQQPFPQQPQQPCPL	0	2	0
PQQQPFPQQPQP	6	0	0
PQQPFQPQQPFPQQPQPY	1	0	0
PLQPQQPFPQPQL	1	0	0
PQQPQQPFPQPQQPFPW	10	0	4
QQPQQPFPQPQQPF	0	0	1
PQQPQQPFPQPQQQLPL	4	0	0
QPQQPFPQQPQQPFPQQPQQIIF	8	0	0
PQQPQQPFPQQPQQIIFQQPQQSY	1	0	0
PQQPQQPFPQQPQQIIF	1	0	0
QQPFPQQPQQPFPQQPQQPL	0	1	0
QPQQPFPQPQEPIPQQPQQPFPQQPQQPF	3	0	0
PQQPQQPFPQQPQQPVPQQPLQQF	3	0	0
PFPQQPQQPFPQPQQPQQPF	5	0	0
QPQQPYPQQPQQPFPQTQQPQQPF	5	0	0
PQQPQQPFPQSQQQCLQQPQHQF	0	1	0
PQQPQQPFPQQTIPQQPQPY	0	3	0
PQQPQQPFPQQSQQPFPQTQQPQQPF	1	0	0
PQQPQQPFPQPQQSFPW	1	0	0
PQQPQQPFPQPQQPQQPFPQL	3	0	0
PQQPQQPFPQPQQL	0	1	0
PQPQQPQQPFPQPQQPQLPF	1	0	0
PQQPQQPFPQQPQQPYPQPQQPQQL	3	0	0
PQQPQQPYPQQPQQPFPQPPQQPY	2	0	0
VQGQGIIQPQQPAQL	0	0	1
GIIQPQQPAQL	0	0	1
PQPQLPYPQPQPF	0	0	1

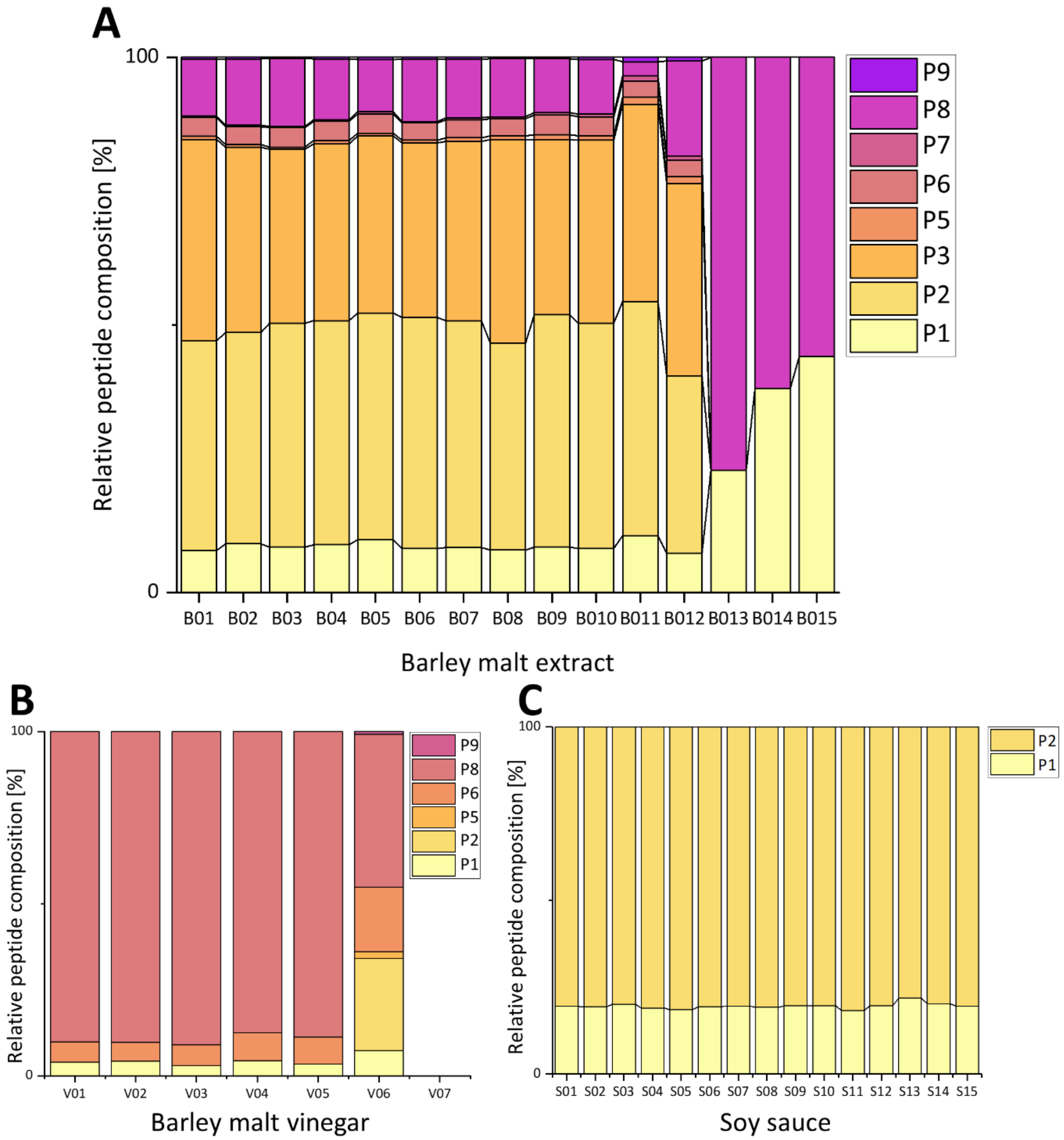

Figure S1: Relative peptide composition considering eight celiac disease-active peptides in barley malt extract (A), barley malt vinegar (B), and soy sauce (C), analyzed by targeted nano-liquid chromatography-tandem mass spectrometry with stable isotope dilution assay.

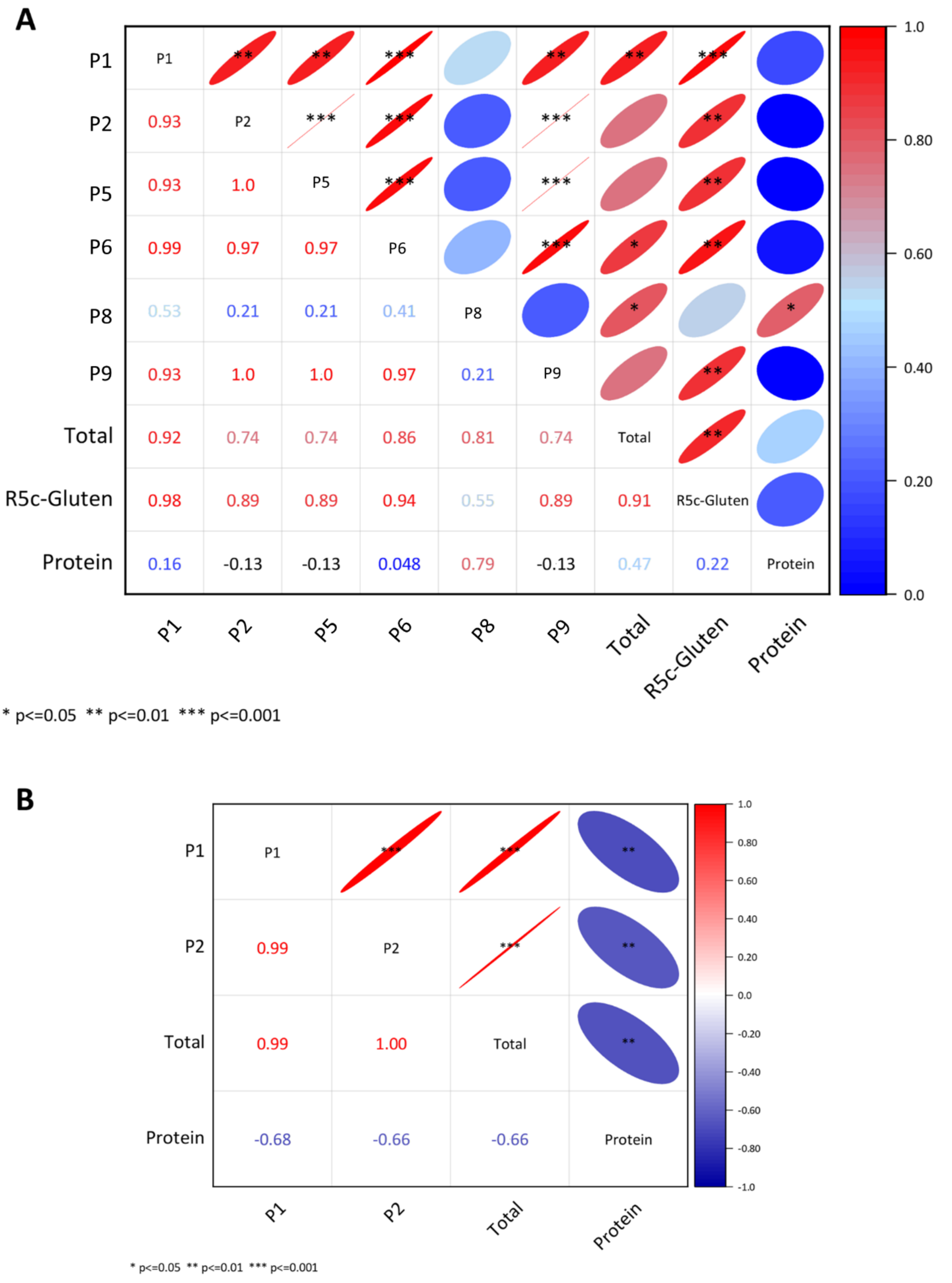

Figure S2: Correlation matrix of the absolute concentration of celiac disease-active peptides P1–P9, the summed total concentration of the peptides, the gluten content, and crude protein content in barley malt vinegar (n = 6) (A) and soy sauce (n = 15) (B). Only parameters where at least one sample obtained values above the limit of quantitation were considered for correlation analysis. Analyses were performed with R5 competitive enzyme-linked immunosorbent assay (R5c ELISA) for R5c-gluten content, Dumas combustion method for protein content and nano-liquid chromatography-tandem mass spectrometry with stable isotope dilution assay for the concentration of peptides P1–P9.

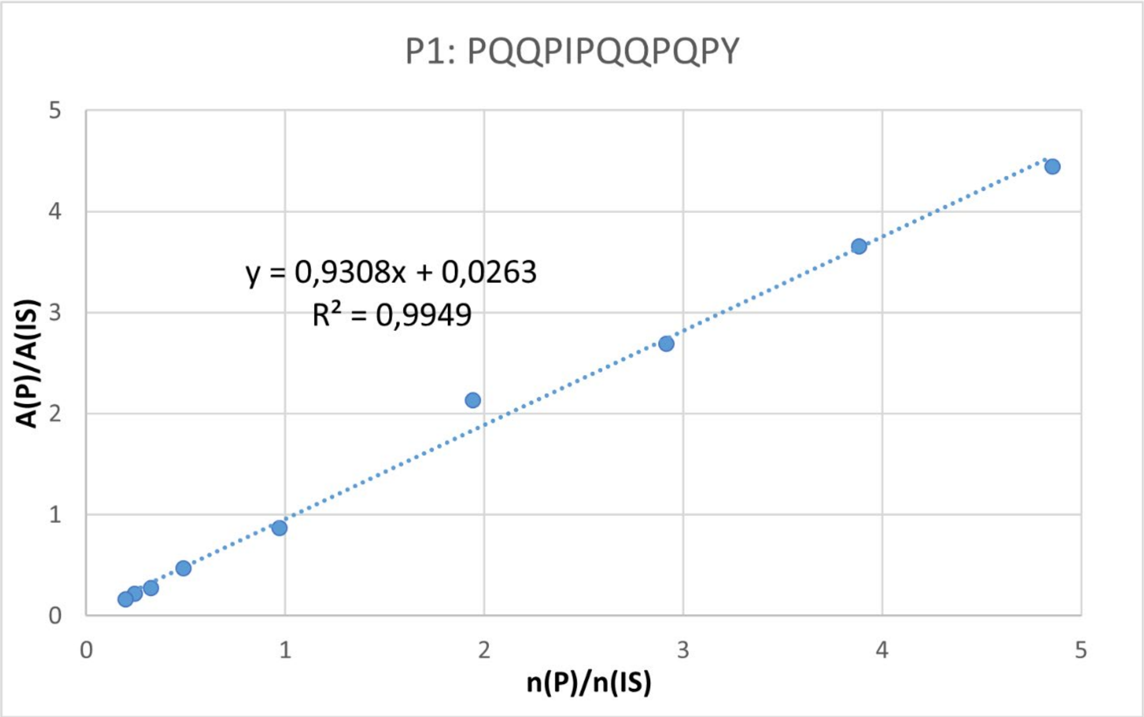

Figure S3: Representative response line of peptide P1. It was used for the absolute quantitation of celiac-disease active peptides across different foods by targeted nano-liquid chromatography-tandem mass spectrometry with stable isotope dilution assay. The line equation for the linear regression is given, as well as the coefficient of determination R^2, determined by plotting the molar ratio of the light peptides (P) and internal standard (IS) n(P)/n(IS) against the peak area ratios A(P)/A(IS). All corresponding raw data has been deposited online (see final link in the main paper).
